# Race, ethnicity, and ancestry reporting in genetic counseling research: A focused mapping review and synthesis

**DOI:** 10.1002/jgc4.1884

**Published:** 2024-02-16

**Authors:** Marta Arpone, Erin Turbitt, Alison McEwen

**Affiliations:** ^1^ Graduate School of Health University of Technology Sydney Ultimo New South Wales Australia; ^2^ Genomic Medicine The Murdoch Children's Research Institute Parkville Victoria Australia; ^3^ Hunter Genetics, NSW Health Waratah New South Wales Australia

**Keywords:** ancestry, disparities, diversity, ethnicity, genetic counseling, race

## Abstract

Studies on the use of Race, Ethnicity, and Ancestry (REA) concepts and terms in genetic research are limited. We aimed to describe the collection, reporting, and use of REA data in genetic counseling research. We undertook a focused mapping review and synthesis of the *Journal of Genetic Counseling* 2021 publications. We used a mapping proforma based on the Race, Ethnicity, And Culture in Health checklist to extract data. Of the 177 screened articles, 132 met our inclusion criteria of reporting primary data about participants. The sample REA characteristics were described in 80 (61%) articles, with 6% providing a definition or conceptualization of the REA term/s used and 23% including a rationale for their study in terms of REA factors. Group labels were most often reported using population descriptors, such as “race,” “ethnicity,” “race/ethnicity,” and “ancestry.” Several group labels were used under different population descriptors. For instance, the group labels “White” and “Asian” were used under all population descriptors. Most studies (79%) ascertained REA characteristics by participants' self‐report. Three (15%) of the 20 qualitative studies mentioned the relevance of the interviewers' REA characteristics in relation to the participants' REA characteristics. Of the 55 quantitative studies, 19 (35%) used REA factors in the data analysis. Of the 80 articles describing the sample REA characteristics, 20% referred moderately or a great deal to any REA factors in the results interpretation, 46% acknowledged the REA factors in the study limitations, and 15% discussed the implications of REA reporting for genetic counseling practice. Our review documents extensive variation in how sample REA characteristics are described and used in genetic counseling research. Our findings provide a baseline against which to evaluate the effects of guidelines and recommendations for the collection, responsible use, and report of participants' REA characteristics in genetic counseling research.

**What is known about this topic**

Differences and inconsistencies in the way clinical genetics professionals and researchers perceive, define, ascertain, and report individuals' Race, Ethnicity and Ancestry (REA) have been documented. To date, no published study has focused on this issue specifically in regard to genetic counseling research practice.

**What this paper adds to the topic**

Our focused mapping review and synthesis documents extensive variation in how sample REA characteristics are currently collected, reported, and used in genetic counseling research published by the Journal of Genetic Counseling. Although most studies described participants' REA characteristics, categorization of group labels under population descriptors were inconsistent and less than half of the studies used REA in the study rationale, data analysis, results interpretation, and discussion of study limitations and clinical implications.

## 1 INTRODUCTION

It is widely recognized that genomic research does not yet reflect the ethnocultural heterogeneity in populations (Sirugo et al., 2019). The vast majority of genetic studies to date predominantly include populations of European ancestry (Popejoy & Fullerton, 2016). Failure to include populations of different ancestries has profound clinical repercussions, reducing generalizability and validity of findings, and increasing the inequality and inequities in genomic medicine services for people of different geographic origins (Edwards et al., 2020; Landry et al., 2018; Ndugga‐Kabuye & Issaka, 2019).

Dedicated initiatives, such as the ClinGen Ancestry and Diversity Working Group in the United States (Clinical Genome Resource, n.d.), the Human Heredity and Health in Africa (H3Africa) (Mulder et al., 2018), and the National Centre for Indigenous Genomics (Australian National University, n.d.) and the Centre for Population Genomics (Garvan Institute of Medical Research and Murdoch Children's Research Institute, n.d.) in Australia are focusing on addressing diversity in genomic medicine and research. Work to scrutinize the terminology and methods used in genomics to describe individuals and populations' Race, Ethnicity and Ancestry (REA) is also underway. The analyses of the collection, report, and use of REA data in genomic research are crucial to develop guidelines that foster research replicability and provision of client‐centered genomic medicine services for diverse populations, and to decrease the likelihood that the inappropriate use of REA data will lead to damaging misconceptions of the relationships between REA and genetics.

Despite these initiatives, there are no comprehensive, widely accepted guidelines for the collection and use of individuals' REA characteristics in research and clinical genetics practice. Different consortia have partnered to address this issue (Popejoy et al., 2018, 2020) but to date, few studies have systematically investigated how REA concepts and terms have been used in genetic research. A pioneering study on the issue documented that although the use of race and ethnicity as variables in genetic research was widespread in the articles analyzed at the time, definitions of these terms were not included and explanations of their use were rarely provided (Sankar et al., 2007).

In the 15 years since Sankar et al. (2007) study, REA concepts, terms, and use in genomics research have continued to be characterized by variation between studies. A contributing factor is that the definitions of race and ethnicity are constantly changing (Flanagin et al., 2021). Their use in health research has also been widely debated (Ali‐Khan et al., 2011; Hunt & Megyesi, 2008; Yudell et al., 2016).

We share the view that race and ethnicity are dynamic social constructs, that are geographically, culturally, and socio‐politically shaped (Borrell et al., 2021), with no biological meaning. Ethnicity refers to a sociopolitical system for classifying people, usually based on their cultural identity (e.g., language, custom, and religion), while race refers to a sociopolitical system classifying and ranking people based on perceived similar ancestral origin and physical attributes (including skin color) (National Academies of Sciences, Engineering, and Medicine, 2023; Stamper, 2019). In contrast, ancestry refers to the country or region of origin or lineage of a person (Flanagin et al., 2021; National Academies of Sciences, Engineering, and Medicine, 2023). A distinction should be highlighted between ancestry as just described and *genetic* ancestry, which is usually inferred based on some genetic similarity measure(s) (Fujimura & Rajagopalan, 2011; National Academies of Sciences, Engineering, and Medicine, 2023). These conceptualizations and definitions are not unanimously embraced; alternatives are offered by experts in various academic fields, health professionals, and lay people. The differences in conceptualization and definition are exemplified by a recent survey, which detected a high rate of inconsistencies in how clinical genetics professionals and researchers perceive, define, and measure REA (Popejoy et al., 2020). The researchers concluded that there is a need for standardization of REA data collection and use in genomic health medicine and research (Popejoy et al., 2020).

The need for standardization, alongside the issues of disparities and racism in research and health care, is also evident in scientific journals. Recommendations and guidelines for authors have been developed (Brothers et al., 2021; Flanagin et al., 2021; Khan et al., 2020, 2022). In 2023, the National Academies of Sciences, Engineering, and Medicine in the United States published a report on the use of REA as population descriptors in genomics research that provides a new framework and decision aid to assist researchers in choosing the most appropriate population descriptors for their study (National Academies of Sciences, Engineering, and Medicine, 2023). To the best of our knowledge, no published study has focused on REA reporting specifically in genetic counseling research.

The language and content of the genetic counseling literature inform genetic counselors' worldviews and clinical practice. To provide a client‐centered genetic counseling service and to conduct research that leads to improved clinical practice for diverse populations, genetic counselors must learn how counselees' health‐care beliefs, values, communication, and decision‐making style, are influenced by their perceived REA background. Yet, it is currently unknown to what extent the cultural safety and conscious awareness about ethnocultural issues advocated in the clinical setting is reflected in genetic counseling research practice.

We aimed to offer an overview of how REA data are collected, reported, and used in genetic counseling research, by conducting a focused mapping review and synthesis examining REA reporting in the *Journal of Genetic Counseling* over the period of 1 year. Specifically, we sought to examine the proportion of articles describing the sample in terms of REA, the terminology used to describe participants' REA characteristics, the methods used for collection of REA data, and the use and reference to REA factors in the study rationale, data analysis, results' interpretation, and discussion of study limitations and clinical implications. Ultimately, the objective of our study was to provide a baseline against which to evaluate the effects of current and future recommendations for the collection, responsible use, and report of participants' REA characteristics in genetic counseling research.

## 2 METHODS

We undertook a focused mapping review and synthesis, examining how REA data are collected, reported, and used in genetic counseling research. To increase depth at the expense of breadth to maximize feasibility, our focus was exclusively on the *Journal of Genetic Counseling*, a prominent and internationally recognized journal in the field, publishing a large number of genetic counseling research articles within a one‐year period.

Our review was informed by the method described as “focused mapping review and synthesis” developed by Bradbury‐Jones et al. (2017) and by other published studies, some investigating race, ethnicity, and culture reporting in allied health research (Engstrand et al., 2018; Lewis‐Fernández et al., 2013; McCambridge & Elkins, 2021) and others focusing on race, ethnicity, and ancestry data use in human genetics research (Ali‐Khan et al., 2011; Sankar et al., 2007).

### 2.1 Search strategy and article selection

The work for this review started in 2021 when the first author was enrolled in a Master of Genetic Counseling program. We included articles published in any of the six issues of the most current volume 30 (year 2021) of the *Journal of Genetic Counseling*. Our inclusion criteria were articles that reported primary data about participants/clients/patients/consumers/members of general public, or health professionals/researchers/educators/students. We excluded articles not reporting primary data.

We identified relevant studies through a two‐stage process. First, two independent reviewers (MA and AM) screened the titles and abstracts of all records against the review eligibility criteria, using the Covidence management software for systematic reviews (Veritas Health Innovation, 2021). Any disagreement regarding the screening was resolved through discussion between MA and AM. To determine final inclusion, MA reviewed the full texts of the included studies. Full texts that were identified to have been erroneously included at the screening stage were excluded from the final dataset upon agreement between MA and AM.

The *Journal of Genetic Counseling* released author guidelines about using inclusive language in September 2021, which also address the use of REA descriptors. We, therefore, verified that all the articles we had included in our review had been submitted for publication prior to the release of these guidelines to serve as a baseline indication of report and use.

### 2.2 Data extraction methods

We charted the results using a mapping proforma. The proforma was developed based on the items of the GAP‐REACH checklist, the Race, Ethnicity, And Culture in Health checklist developed by the Cultural Committee of the Group for the Advancement of Psychiatry (GAP) (Lewis‐Fernández et al., 2013), which has also been applied elsewhere (Engstrand et al., 2018). The variables included in the mapping proforma are presented in Table 1, whereas Table S1 reports the instructions for the data extraction for each variable.

**TABLE 1:** Mapping proforma items.

*Item*	
1.	Article title
2.	Author list
3.	Specialty
4.	Study aim/s
*REA definition and study rationale*	
5.^a^	Define REA factor(s)
6.^a^	Discuss the role of REA factors in the rationale for the study
*Methods and results*	
7.	Study design/method
8.	Country/ies where the study took place
9.	Characteristics of the sample
10.^a^	Include REA factors in sampling procedure
11.^a^	Describe REA characteristics of the sample
12.^a^	Describe how participants' REA characteristics were ascertained
13.	Specific method of REA characteristics ascertainment in case of self‐report
14.	REA population descriptors
15.	Group labels used to describe the sample
16.	Report “Other” among group labels
17.^a^	Mention match or mismatch of interviewers' and participants' REA characteristics
18.^a^	Use REA factors in data analysis
19.	Perform “White” versus “non‐White/non‐Caucasian/Other/more than once race” data analysis
*Discussion*	
20.^a^	Refer to REA factors in the interpretation of results
21.^a^	Include REA factors in the discussion of study limitations
22.^a^	Discussion of implications of REA reporting for genetic counseling practice

^a^ Indicates items for which inter‐coder reliability was analyzed.

We undertook data extraction for each article that met eligibility criteria by reviewing the full texts, including any relevant additional attachments. Two researchers (MA and AM) independently extracted information from 28 (21%) randomly selected full‐text articles. The data independently extracted by the two researchers from the first 14 articles were compared and discussed, and instructions for data extraction were refined to maximize clarity. Then, a further 14 full‐text articles were independently used for data extraction. We investigated the inter‐coder reliability of the data extracted from all 28 articles for the variables for which data were more ambiguous and deemed more likely to be affected by the coders' appraisal (indicated with an asterisk in Table 1) by calculating Cohen's Kappa coefficients (*k*; Landis & Koch, 1977) with 95% confidence interval (CI). Data extraction and data entry from the remaining articles was performed independently by MA. Validation of a subset of this data extraction was undertaken by AM. Approximately 12 months after the first data extraction, MA re‐extracted data from 30% of articles reporting participants' REA characteristics in order to ascertain intra‐coder reliability.

### 2.3 Data analysis and results presentation

We summarized descriptive results for categorical variables (e.g., number of articles reporting REA data) with counts and percentages. We analyzed associations between categorical variables of interest (e.g., description of participants' REA characteristics and study design) using chi‐square or Fisher's exact test; *p*‐values (*p*) < 0.05 were considered significant. All statistical analyses were performed using Stata version 13 (http://www.stata.com).

## 3 RESULTS

Of the 177 articles published by the *Journal of Genetic Counseling* in 2021, seven were removed before screening (six were issue information and one was a list of reviewers), 38 were excluded for not reporting primary data, and 132 met our inclusion criteria. A list of the 132 articles included in the review is provided in Appendix S1: Note 1.

The inter‐coder reliability calculated on the data extracted from the first 28 articles varied from *almost perfect* for the “description of the sample REA characteristics” variable (100%; *k* = 1.00; SE = 0.189) to *fair* for the “discussion of the study rationale in terms of REA factors” (67.86%; *k* = 0.292; SE = 0.184). Inter‐coder reliability details for the remaining variables ranged from *moderate* to *almost perfect* (Appendix S1: Note 2). AM performed data validation on the data independently extracted by MA from six additional articles: for the above‐mentioned variables, and the variables included in Appendix S1: Note 2, agreement was perfect (100%). Intra‐coder reliability analyses revealed that 85% of the articles, from which data extraction was repeated at a second time point, had 100% data consistency across all variables.

### 3.1 General characteristics of the studies

The 132 articles covered a wide range of genetic counseling topics, from cancer and pediatric genetics to cross‐disciplinary research (for further details see Table S2). The studies included primary data collected most frequently from North America (75%), followed in order of frequency by Europe, Australasia, Asia, Africa, and the Middle East (Table S3). Most of the studies used a quantitative design (*n* = 86; 65%), whereas qualitative and mixed‐methods designs were adopted by 39 (30%) and 7 (5%) studies, respectively. The data used in these studies were collected from (i) individuals from the general population or patients (or their samples/medical records) (*n* = 80; 61%), and (ii) health professionals (e.g., genetic counselors and geneticists), genetic counseling students or educators (*n* = 49; 37%). Three (2%) studies reported data collected from both the above‐mentioned groups (Table S4).

### 3.2 REA characteristics of the study samples and REA factors in study design

The REA characteristics of the sample included in the studies were described in 80 (61%) articles. Of these 80 articles, 18 (23%) provided a rationale for their study in terms of REA factors and five (6%) included a definition or conceptualization of the REA term/s used (Table S4).

The proportion of studies that described the sample REA characteristics did not differ by study design (quantitative (56/86; 65%) vs. qualitative (20/39; 51%), *X* ^2^ (1, *N* = 125) = 2.15, *p* = 0.142). However, a higher proportion of articles reporting on patients/ general population (58/80; 73%) described the sample REA characteristics compared to articles on health professionals/students (21/49; 43%, *X* ^2^ (1, *N* = 129) = 11.25, *p* = 0.001) (Table S4). Of the 80 articles reporting the sample REA characteristics, 12 (15%) attended specifically to REA factors in sampling (e.g., justified sampling a single REA group or stratified the sample by REA groups) (Table S4).

### 3.3 Population descriptors and group labels used to describe the REA characteristics of the sample

**FIGURE 1:**
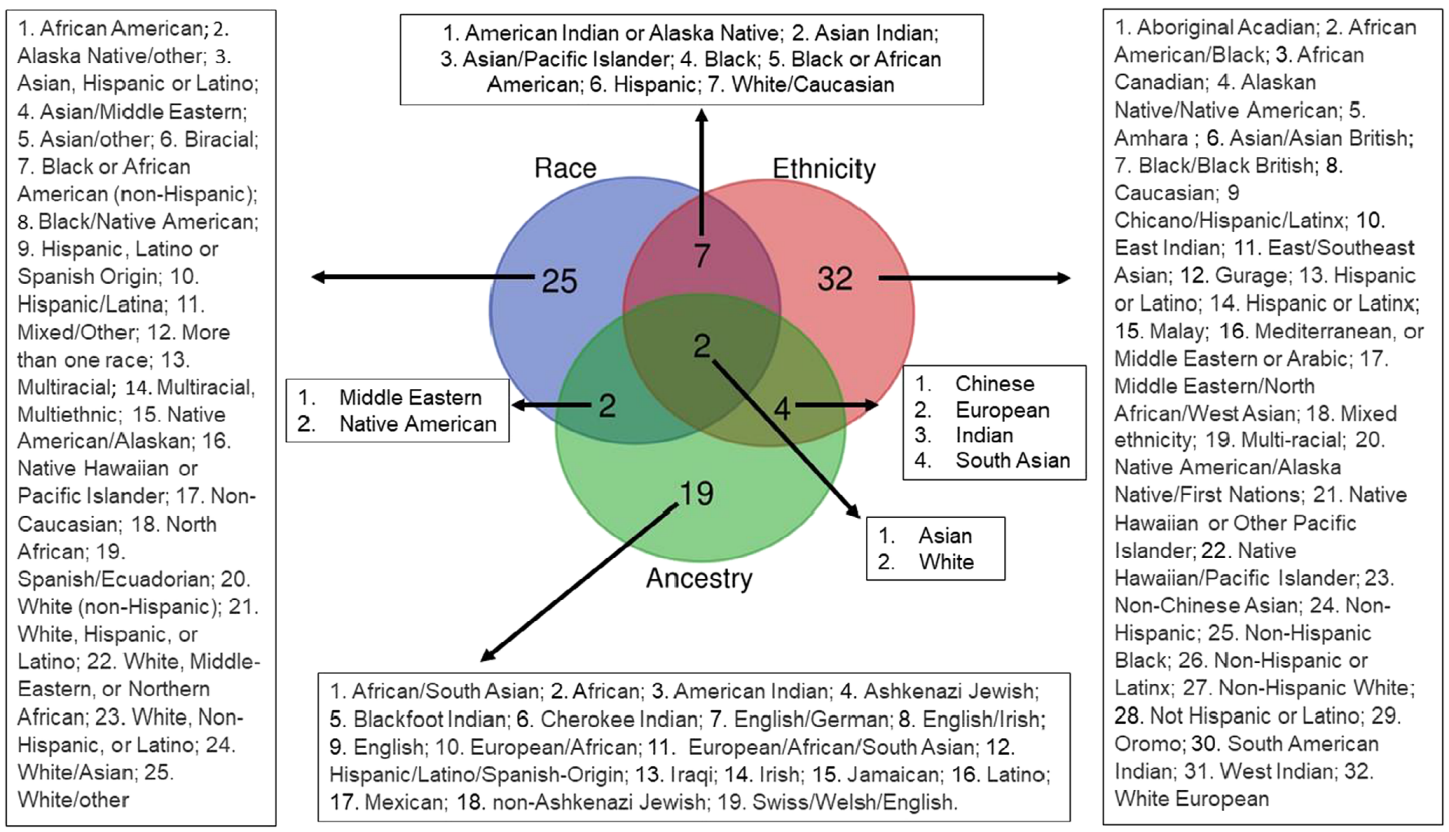
Venn diagram of REA population descriptors and group labels used for sample description.

Of the 80 articles describing the sample REA characteristics: 43 (54%) used either race or ethnicity as a population descriptor, or both population descriptors but with separate group labels (e.g., race: “White,” “Asian”; and ethnicity: “Not Hispanic or Latino,” “Hispanic or Latino”), 20 (25%) articles used a combined race and ethnicity population descriptor (e.g., "race/ethnicity" or "race and ethnicity"), 4 (5%) articles used “ancestry,” whereas in 13 (16%) articles the population descriptors were not specified. Analysis of the participant multiple‐choice surveys provided as additional attachments allowed the identification of few differences in the terminology used in the questions posed to the participants and in the study results. The group labels used in the study results to describe the REA characteristics of the sample, stratified by the different assigned population descriptors (i.e., “race,” “ethnicity,” and “ancestry”), is presented in Figure 1. The number of group labels used exclusively under each of these three population descriptors was 25, 32, and 19, respectively. Conversely, some group labels were used under different population descriptors across articles. For instance, the group labels “Middle Eastern” and “Native American” were used under both the race and ancestry population descriptors (Figure 1). The 20 articles that used a combined race and ethnicity population descriptor included the group labels listed in Appendix S1: Note 3.

In other articles, group labels, such as “Latinas,” “Omani Muslims of Arab descents,” and “Black South African,” were reported but not categorized explicitly by the authors as race, ethnicity, or ancestry. Lastly, 28 articles included “other” among the REA group labels.

### 3.4 REA characteristics ascertainment method

Most studies (63/80; 79%) ascertained REA characteristics by participants' self‐report (Table S4). However, for 37/63 (59%) of these studies, the type of self‐report method was not clearly specified. Of the remaining 26 studies, one elicited demographic information through a telephone survey (Lynce et al., 2021) and the other 25 studies used a multiple‐choice survey method, with or without the option for participants to include a REA description in their own words.

Six (8%) studies extracted REA characteristics from the medical records while in one study (1%), the researchers assigned a “racial/ethnic” group label based on the reported country of ancestral origin (Isley et al., 2021). Of the 80 articles reporting the REA characteristics of the sample, 10 (13%) did not describe the REA characteristics ascertainment method.

### 3.5 REA characteristics of interviewers and interviewees

The relevance of the REA characteristics of the interviewers in relation to the participants' REA characteristics was mentioned in 15% of the 20 qualitative studies and 0% of the three mixed‐methods studies that conducted interviews or focus groups and reported the participants' REA characteristics (Table S4).

### 3.6 Use of REA factors in data analysis

Of the 56 quantitative studies reporting the participants' REA characteristics, 55 included more than one REA group or recruited participants from a specific REA group with the intent of studying it (i.e., not a convenience sample resulting in a homogenous group of participants with the same REA characteristics). Nineteen (35%) of the 55 studies used the REA factors in the data analysis (Table S4). Of these 19 studies, among those including more than one REA group, five performed analyses comparing “White” or “White Caucasian” participants versus a group described as “more than one race” or “Other” or “non‐White” or “non‐Caucasian,” where the "non‐White" or "non‐Caucasian" group labels encompassed participants of different racial, ethnic, or ancestral background (Table S4).

### 3.7 Consideration of REA factors in the studies' discussion

Of the 80 articles reporting the participants' REA characteristics, 16 (20%) referred moderately or a great deal to any REA factors in the interpretation of their results. Thirty‐seven (46%) acknowledged the REA factors in the study limitations discussion but only 12 (15%) discussed the implications of REA reporting for genetic counseling practice (Table S4).

Among the studies that described participants' REA, those that provided a rationale for the study design in terms of REA factors, in comparison to those that did not provide such a rationale, were more likely to have: interpreted the results taking into consideration participants' REA characteristics (11/18 vs. 5/62; *p* = <0.001), discussed the limitations of the study in terms of REA (15/18 vs. 22/62; *p* < 0.001) and discussed REA reporting implications for genetic counseling practice (9/18 vs. 3/62; *p* < 0.001).

## 4 DISCUSSION

We describe the collection, reporting, and use of REA data in genetic counseling research published by the *Journal of Genetic Counseling* in 2021. Most studies described participants' REA characteristics. However, categorization of group labels under REA population descriptors was inconsistent across publications and less than half of the studies took REA factors into account in the study rationale, data analysis, results interpretation, and discussion of study limitations and clinical implications.

The most commonly reported method for REA data collection was participant self‐report via multiple‐choice surveys. Collection of self‐reported race and ethnicity using only multiple‐choice questionnaire with pre‐defined responses reduces data variability, in fact one article included in this review explicitly highlighted that “when asked to describe their race or ethnicity in their own words, participants offered a much broader range of categories” (Carmichael et al., 2021, p. 816; see Appendix S1: Note 4 for further details).

Ancestry ascertainment via participants' self‐report also has limitations including the possible issue of self‐reported ethnicity as a proxy for ancestry. The National Academies of Sciences, Engineering, and Medicine (2023) has in fact pointed out that labels referring to geography, ethnicity, or race are frequently used to designate groups whose members are supposed to share common ancestry. The findings from our review, such as the use of the group labels African, Asian, and White to describe participants' ancestry, are consistent with this statement.

These issues highlight the need to scrutinize the necessity and rationale of collecting REA information from research participants and the need to define the terminology used, not only in the published articles but also in the participant questionnaires so that ambiguity can be reduced. Further research to ascertain culturally appropriate and consistent methods to collect standardized REA data is needed.

The REA characteristics of the study participants were described in 61% (*n* = 80) of included articles, with a higher frequency in studies of patients/members from the public than of health professionals/students. These findings suggest that genetic counselors may be more prone to turn their lens onto “others” than onto themselves, possibly because they recognize that the profession lacks the diversity of the client group they serve. In 2021, the US National Society of Genetic Counselors administered a professional status survey to its members and 90% of the responders identified as White (NSGC Professional Status Survey: Executive Summary, 2021). An Australian census of clinical genetic health‐care professionals did not report any REA characteristics of the survey responders (Nisselle et al., 2019). The lack of researchers' and health professionals' REA characteristics was evident in the reflexive statements of the qualitative studies included in this review. Only a few studies mentioned the relevance of the REA characteristics of the interviewers in relation to the participants' REA characteristics. One of the ways in which the profession may increase diversity (Bonham & Green, 2021) is by demonstrating a willingness to collect and report REA data so that diversity can be tracked over time.

A small proportion of studies provided a definition or conceptualization of the REA term/s used and a rationale for their study in terms of REA factors. Similar results were obtained in a previous study that analyzed 330 randomly selected genetic‐related articles published between 2001 and 2004 (Sankar et al., 2007), which found that no article offered a definition of race and ethnicity and only a few explained how describing the race or ethnicity of the study population was relevant to address the study research question. Recent recommendations from the US National Academies of Sciences, Engineering, and Medicine state that genetics and genomics researchers should provide a rationale for their chosen population descriptors. This recommendation (along with others in the report) may be applicable to genetic counseling researchers (National Academies of Sciences, Engineering, and Medicine, 2023).

The lack of definitions might be underlined by authors assuming that readers, as well as other researchers, have univocal definitions of these terms. The findings of our review, as illustrated by Figure 1 would confute this assumption.

Our review also highlights frequent use of the combined phrases “race/ethnicity” and “race and ethnicity”. On one hand this terminology is in line with proposals of unifying these terms in aggregate categories (Flores, 2020) due to discrimination concerns (González‐Hermoso & Santos, 2019). On the other hand, though, merging race and ethnicity in one single “race/ethnicity” population descriptor is no longer recommended (Flanagin et al., 2021) as this terminology blurs the distinction between the two concepts and may potentially lead to misunderstanding about the relationships between race and ethnicity, and genetic counseling research findings. Guidelines about how to harmonize the use of these terms will help reduce the inconsistencies between studies.

The findings of our review suggest a form of tokenism in collecting and using participants' REA data, where tokenism is intended as a symbolic practice rather than a thoughtful and purposeful research effort. As such, the findings substantiate the concern already highlighted by Sankar et al. (2007), according to which genetics researchers not only do not provide definitions of race, ethnicity, and ancestry but also do not explain the relevance of these variables to their research. The need to move toward the meaningful practice of gathering and purposefully using REA data, which is advocated in the literature addressing these topics (e.g., Flanagin et al., 2021; Hindorff et al., 2018; Khan et al., 2020; Lee et al., 2008), seems largely untranslated in current genetic counseling research practice.

A discussion of when participants' REA reporting could or could not be considered relevant and appropriate to the research questions addressed by the individual studies is beyond the scope of the current review as we sought to document, rather than evaluate, research reported in 2021. However, we hope the results from our study will prompt further reflection on how, when, and why we should collect, use, and report REA information in genetic counseling research.

### 4.1 Study limitations

This review is constrained by several limitations. We focused on a single journal and therefore, results are not generalizable to the broader genetic counseling research field. Similarly, the results may not reflect worldwide genetic counseling research practice as the evidence upon which the results are based primarily originates from research studies conducted in the USA. This is unsurprising considering that out of the estimated 7,000 genetic counselors working globally about 4,000 practice in this country (Abacan et al., 2019) and that the *Journal of Genetic Counseling* is published in partnership with the US National Society of Genetic Counselors.

We also acknowledge the interpretative rather than factual nature of some the findings we reported, as the data extracted for some variables (e.g., extent of the reference to REA factors in the results interpretation) was based on our subjective evaluation. Notably, despite extensive discussions between the data extractors, inter‐rater reliability for some variables remained suboptimal, underlining the inherent ambiguity in the material and the relative subjectivity in researchers' appraisal of the use of, and reference to, REA data. Lastly, this study did not address the associations between studies' topics and the variables of interest nor proposed a qualitative analysis of data, such as the stated purpose for REA data collection and the described implications of REA reporting for provision of genetic counseling services.

### 4.2 Practice and research implications

The limited attention devoted to REA reporting and use in the genetic counseling research field may have flow‐on effects in the clinical practice setting, as these realms are inherently intertwined. A consistent and considerate research approach would foster the translation of research evidence into clinical practice and facilitate equitable access to genomic medicine and effective genetic counseling for diverse populations. For instance, lack of, inconsistent, or non‐specific use of population descriptors and group labels in research can have detrimental effects in clinical settings. It can exacerbate the challenges of developing, and clinically implementing, tailored genetic counseling interventions, especially for underrepresented communities, and potentially lead to, or fail to help address, genetic counselors' unconscious prejudice. The awareness of the universality of implicit biases requires genetic counseling researchers to exercise reflexivity in their research practice. As an example, study results dichotomized in “White” versus “non‐White,” a practice known as othering, could have deleterious repercussions. This methodological approach does not foster the predominantly White genetic counseling workforce's cultural competence, which is essential to serve a diverse clientele. In addition, it also carries the risk of creating a discourse of separation and distancing between “Us” and “Them” (Grove & Zwi, 2006), which would in turn hinder the working alliance and rapport between counselors and clients.

The quality of provision of patient‐centered, culturally sensitive, and effective genetic counseling services is impacted by the narratives of the genetic counseling scientific community. It has been posited that the lack of careful use and communication of REA concepts in publications can lead to harmful misconceptions of these constructs and their relationship to genetics (Khan et al., 2020, 2022). This in turn can lead to biologically reify social concepts (Braun, 2006), such as race, and consequently to fuel racism (Lee et al., 2008). Language shapes individuals' worldviews and thinking processes. The terminology and vocabulary used to describe people involved in genetic counseling research, and the conceptualization, relevance, and use of REA factors in this research setting are crucially important.

Consistencies in REA information collection, use, and reporting in research would also support the production of evidence that is valid, replicable, and comparable across studies. Our review method and study findings may inform future similar reviews on REA data collection, report, and use in genetic counseling research studies published by other scientific journals and by the *Journal of Genetic Counseling* in future years. In September 2021, after all the articles included in this review had been accepted for publication, the *Journal of Genetic Counseling* made available, in its online author guidelines, resources for authors' use of inclusive language (The Journal of Genetic Counseling, 2021). It would be of interest to replicate our study in the future to study the effect of those guidelines and those published in the report by the National Academies of Sciences, Engineering, and Medicine (2023) on the REA data collection, use and reporting in the journal.

A deeper and broader insight of the current genetic counseling research practice regarding participants' REA data collection and use is indeed warranted. Future studies could also offer a narrative thematic synthesis of qualitative data, such as the stated relevance of REA factors in the study rationale and discussion of implications of REA reporting for genetic counseling practice. This approach could provide contextual information to the quantitative results obtained in the current and future studies. Lastly, survey and qualitative studies involving researchers, advocates, and other interested stakeholders would also be valuable to understand their perspectives about how population descriptors and group labels are currently used in genetic counseling research, how they could be improved, and what approach might be used effectively in the future.

### 4.3 Conclusion

Our review and synthesis document extensive variation in how genetic counseling research studies describe their sample REA characteristics. REA reporting seems in most cases to constitute a token research task rather than a contextualized reflection and discussion of REA factors in relation to the study aim, research questions, results, and clinical implications. Our findings highlight the need for initiatives that could improve genetic counseling research practice. We provide a baseline map against which to evaluate journals' and researchers' adherence to guidelines and recommendations for the collection, responsible use, and report of participants' REA information in genetic counseling research.

## AUTHOR CONTRIBUTIONS

**Marta Arpone**: conceptualization, methodology, investigation, formal analysis, visualization, writing—original draft, writing—review and editing; **Erin Turbitt**: conceptualization, methodology, supervision, writing—review and editing; **Alison McEwen**: conceptualization, methodology, investigation, validation, supervision, writing—review and editing. Marta Arpone confirms to have full access to all the data in the study and take responsibility for the integrity of the data and the accuracy of the data analysis. All of the authors gave final approval of this version to be published and agree to be accountable for all aspects of the work in ensuring that questions related to the accuracy or integrity of any part of the work are appropriately investigated and resolved.

## CONFLICT OF INTEREST STATEMENT

Marta Arpone, Erin Turbitt and Alison McEwen declare that they have no conflict of interest.

## ETHICS STATEMENT

Human studies and Informed Consent: This study did not involve human subjects.

Animal Studies: No non‐human animal studies were carried out by the authors for this article.

## Supporting information


Appendix S1

## Data Availability

The data that support the findings of this study are available from the corresponding author, upon reasonable request.
