# Supplementary material for: Race, ethnicity, and ancestry reporting in genetic counseling research: A focused mapping review and synthesis
Source: J Genet Couns. 2024 Feb 16;34(1):e1884. doi: 10.1002/jgc4.1884 (PMC11726610; doi:10.1002/jgc4.1884)
Supplement: Supplementary file 1 — Appendix S1 [file JGC4-34-0-s001.docx]

**Race, ethnicity and ancestry reporting in genetic counseling research: a focused mapping review and synthesis**

Arpone, Turbitt, McEwen

**Supplemental Note 1: Reference list of articles included in the review**

Aamodt, Pauline, Wetherill, Leah, Delk, Paula, Torres-Martinez, Wilfredo, Vance, Gail H., & Wesson, Melissa. (2021). Positive and negative professionalism experiences of genetic counseling students in the United States and Canada. *Journal of Genetic Counseling, 30*(2), 478-492. doi:https://doi.org/10.1002/jgc4.1334

Aeilts, Amber M., Carpenter, Kristen M., Hovick, Shelly R., Byrne, Lindsey, Shoben, Abigail B., & Senter, Leigha. (2021). The impact of a cascade testing video on recipients’ knowledge, cognitive message processing, and affective reactions: A formative evaluation. *Journal of Genetic Counseling, 30*(3), 656-664.

doi:https://doi.org/10.1002/jgc4.1345

Agre, Katherine, Essendrup, Anna, Koellner, Christine, Rust, Laura, & Deyle, David. (2021). Examining career shadowing in genetic counseling: Perspectives of shadowees, program directors, and genetic counselors. *Journal of Genetic Counseling, 30*(5), 1428- 1439. doi:https://doi.org/10.1002/jgc4.1411

Al-Kharusi, Khalsa, Bruwer, Zandre, & Wessels, Tina-Marié. (2021). The experience of Preimplantation Genetic Testing (PGT) among Muslim couples in Oman in the Middle East. *Journal of Genetic Counseling, 30*(1), 121-131.

doi:https://doi.org/10.1002/jgc4.1300

Aldridge, Caitlin E., Osiovich, Horacio, Siden, Harold, Study, Rapidomics, Gen, Counsel Study, & Elliott, Alison M. (2021). Rapid genome-wide sequencing in a neonatal intensive care unit: A retrospective qualitative exploration of parental experiences. *Journal of Genetic Counseling, 30*(2), 616-629. doi:https://doi.org/10.1002/jgc4.1353

Allen, Caitlin G., Ritchie, Jordon B., Morrison, Heath, Lauzon, Steven D., Nichols, Michelle, Schiffman, Joshua D., . . . Welch, Brandon M. (2021). A thematic analysis of healthinformation technology use among cancer genetic counselors. *Journal of Genetic Counseling, 30*(1), 170-179. doi:https://doi.org/10.1002/jgc4.1306

Alvarado-Wing, Tatiana E., Marshall, Jennifer, Best, Alicia, Gomez, Joanne, & Cragun, Deborah. (2021). Exploring racial and ethnic minority individuals’ journey to becoming genetic counselors: Mapping paths to diversifying the genetic counseling profession. *Journal of Genetic Counseling, 30*(6), 1522-1534.

doi:https://doi.org/10.1002/jgc4.1419

Arjunan, Aishwarya, Sanders, Merideth, Chen, Anthony, Roscow, Breanna, & Ray, Jessica. (2021). Genetic counseling student rotations in industry: How COVID-19 magnified the urgency for virtual learning options in diverse training settings. *Journal of Genetic Counseling, 30*(5), 1316-1324. doi:https://doi.org/10.1002/jgc4.1500

Aryamvally, Anjali, Myers, Melanie F., Huang, Taosheng, Slone, Jesse, Pilipenko, Valentina, & Hartmann, Julianne E. (2021). Mitochondrial replacement therapy: Genetic counselors' experiences, knowledge, and opinions. *Journal of Genetic Counseling, 30*(3), 828-837. doi:https://doi.org/10.1002/jgc4.1382

Bartley, Nicci, Best, Megan, & Butow, Phyllis. (2021). Pursuing germline genome sequencing to reduce illness uncertainty may involve additional uncertainties for cancer patients: A mixed-methods study. *Journal of Genetic Counseling, 30*(4), 1143-1155.

doi:https://doi.org/10.1002/jgc4.1398

Bergner, Amanda L., Ecker, Lindsey Alico, Ernst, Michelle E., Goelz, Monika Zak, Habermann, Kristina, Karger, Lisa, & Zinberg, Randi E. (2021). The evolution of genetic counseling graduate education in New York City during the COVID-19 pandemic: In the eye of the storm. *Journal of Genetic Counseling, 30*(4), 1057-1068. doi:https://doi.org/10.1002/jgc4.1461.

Boardman, Felicity K. (2021). Attitudes toward population screening among people living with fragile X syndrome in the UK: ‘I wouldn’t wish him away, I’d just wish his fragile X syndrome away’. *Journal of Genetic Counseling, 30*(1), 85-97. doi:https://doi.org/10.1002/jgc4.1355

Bonine, Shea, Bell, Megan, Fishler, Kristen, Berninger, Taylor, & Erickson, Lindsay. (2021). Conscience clauses in genetic counseling: Awareness and attitudes. *Journal of Genetic Counseling, 30*(5), 1468-1479. doi:https://doi.org/10.1002/jgc4.1414

Boothe, Emily, Greenberg, Samantha, Delaney, Christine L., & Cohen, Stephanie A. (2021). Genetic counseling service delivery models: A study of genetic counselors’ interests, needs, and barriers to implementation. *Journal of Genetic Counseling, 30*(1), 283-292. doi:https://doi.org/10.1002/jgc4.1319

Brown, Courtney, Head, Katharine J., Hartsock, Jane, Burns, Katelyn, Wilson, Theodore E., & Prucka, Sandra. (2021). Exploring parents’ perceptions of the value of pediatric genetic counseling patient letters: A qualitative study presenting lessons learned. *Journal of Genetic Counseling, 30*(4), 1168-1180. doi:https://doi.org/10.1002/jgc4.1400

Burke, Sarah, Mork, Maureen, Qualmann, Krista, Woodson, Ashley, Jin Ha, Min, Arun, Banu, & Kaulfus, Meagan. (2021). Genetic counselor approaches to BRCA1/2 direct-to- consumer genetic testing results. *Journal of Genetic Counseling, 30*(3), 803-812. doi:https://doi.org/10.1002/jgc4.1380

Carlotti, Katherine, Hines, Karrie, Weida, Jennifer, Lah, Melissa, & Schwantes-An, Tae-Hwi. (2021). Perceived barriers to paternal expanded carrier screening following a positive maternal result: To screen or not to screen. *Journal of Genetic Counseling, 30*(2), 470-

477. doi:https://doi.org/10.1002/jgc4.1333

Carmichael, Nikkola, Redlinger-Grosse, Krista, & Birnbaum, Shira. (2021). Supporting a sense of inclusion and belonging for genetic counseling students who identify as racial orethnic minorities. *Journal of Genetic Counseling, 30*(3), 813-827. doi:https://doi.org/10.1002/jgc4.1381

Chan, Priscilla A., Lewis, Katie L., Biesecker, Barbara B., Erby, Lori H., Fasaye, Grace-Ann, Epps, Sandra, . . . Turbitt, Erin. (2021). Preferences for and acceptability of receiving pharmacogenomic results by mail: A focus group study with a primarily African- American cohort. *Journal of Genetic Counseling, 30*(6), 1582-1590. doi:https://doi.org/10.1002/jgc4.1424

Cherny, Sara, Olson, Rachael, Chiodo, Kathryn, Balmert, Lauren C., & Webster, Gregory. (2021). Changes in genetic variant results over time in pediatric cardiomyopathy and electrophysiology. *Journal of Genetic Counseling, 30*(1), 229-236. doi:https://doi.org/10.1002/jgc4.1313

Clark, Cheyla R., Reyes, Kathryn, Ormond, Kelly E., Caleshu, Colleen, & Moscarello, Tia. (2021). U.S. Genetic counselors’ perceptions of inpatient genetic counseling: A valuable model for medically complex patients. *Journal of Genetic Counseling, 30*(6), 1683-1694. doi:https://doi.org/10.1002/jgc4.1435

Conijn, Thirsa, Nijmeijer, Stephanie C. M., Lakeman, Phillis, Henneman, Lidewij, Wijburg, Frits A., & Haverman, Lotte. (2021). Preconception expanded carrier screening: Impact of information presented by text or video on genetic knowledge and attitudes. *Journal of Genetic Counseling, 30*(2), 457-469. doi:https://doi.org/10.1002/jgc4.1332

Conley, Claire C., Castro-Figueroa, Eida M., Moreno, Laura, Dutil, Julie, García, Jennifer D., Burgos, Carolina, . . . Vadaparampil, Susan T. (2021). A pilot randomized trial of an educational intervention to increase genetic counseling and genetic testing among Latina breast cancer survivors. *Journal of Genetic Counseling, 30*(2), 394-405. doi:https://doi.org/10.1002/jgc4.1324

Costa, Taylor, Gillies, Brittany, Oh, Tracey, & Scott, Jenna. (2021). The Canadian genetic counseling workforce: Perspectives from employers and recent graduates. *Journal of Genetic Counseling, 30*(2), 406-417. doi:https://doi.org/10.1002/jgc4.1326

Cragun, Deborah, Weidner, Anne, Tezak, Ann, Clouse, Kate, & Pal, Tuya. (2021). Family communication of genetic test results among women with inherited breast cancer genes. *Journal of Genetic Counseling, 30*(3), 701-709. doi:https://doi.org/10.1002/jgc4.1356

Dalpé, Gratien, Pinkesz, Miriam, Oliviero, Elisabeth, Tolymbek, Maria, & Joly, Yann. (2021). Genetic discrimination views in online discussion forums: Perspectives from Canadian forumites. *Journal of Genetic Counseling, 30*(6), 1613-1628.

doi:https://doi.org/10.1002/jgc4.1427

Davis, Dillon, Oakes, Lisa, Raol, Nikhila, Mighion, Lindsey, & Dedhia, Kavita. (2021). Evaluating parental genetic knowledge of hearing loss with and without pre-test genetic counseling in a pediatric otolaryngology clinic. *Journal of Genetic Counseling, 30*(5), 1379-1387. doi:https://doi.org/10.1002/jgc4.1404

De Simone, Lenika M., Arjunan, Aishwarya, Vogel Postula, Kristen J., Maga, Tara, & Bucheit, Leslie A. (2021). Genetic counselors’ perspectives on population-based screening for BRCA-related hereditary breast and ovarian cancer and Lynch syndrome. *Journal of Genetic Counseling, 30*(1), 158-169. doi:https://doi.org/10.1002/jgc4.1305

Desine, Stacy, Eskin, Lena, Bonham, Vence L., & Koehly, Laura M. (2021). Social support networks of adults with sickle cell disease. *Journal of Genetic Counseling, 30*(5), 1418- 1427. doi:https://doi.org/10.1002/jgc4.1410

Deuitch, Natalie T., Beckman, Erika, Halley, Meghan C., Young, Jennifer L., Reuter, Chloe M., Kohler, Jennefer, . . . Tabor, Holly K. (2021). “Doctors can read about it, they can know about it, but they've never lived with it”: How parents use social media throughout the diagnostic odyssey. *Journal of Genetic Counseling, 30*(6), 1707-1718. doi:https://doi.org/10.1002/jgc4.1438

Ding, Huanghe, Sutton, Arnethea L., Hurtado-de-Mendoza, Alejandra, & Sheppard, Vanessa

B. (2021). The role of psychosocial factors in Black women's self-efficacy in receiving genetic counseling and testing. *Journal of Genetic Counseling, 30*(6), 1719-1726. doi:https://doi.org/10.1002/jgc4.1439

Dratch, Laynie, Paul, Rachel A., Baldwin, Aaron, Brzozowski, Morgan, Gonzalez-Alegre, Pedro, Tropea, Thomas F., . . . Bardakjian, Tanya. (2021). Transitioning to telegenetics in the COVID-19 era: Patient satisfaction with remote genetic counseling in adult neurology. *Journal of Genetic Counseling, 30*(4), 974-983.

doi:https://doi.org/10.1002/jgc4.1470

Dugger, Chloe, Anderson, Hannah S., Miller, Christine E., Wong, Bob, Johnson, Erin P., & Rothwell, Erin. (2021). Assessing clinical education tools for expanded carrier screening. *Journal of Genetic Counseling, 30*(2), 606-615.

doi:https://doi.org/10.1002/jgc4.1349

Dwyer, Andrew A., Au, Margaret G., Smith, Neil, Plummer, Lacey, Lippincott, Margaret F., Balasubramanian, Ravikumar, & Seminara, Stephanie B. (2021). Evaluating co-created patient-facing materials to increase understanding of genetic test results. *Journal of Genetic Counseling, 30*(2), 598-605. doi:https://doi.org/10.1002/jgc4.1348

Ehrenberg, Sarah, Walsh Vockley, Catherine, Nelson, Erica, Baker, Jennifer, Arcieri, Michael, Lindenberger, Jessica, & Ghaloul-Gonzalez, Lina. (2021). Under-referral of Plain community members for genetic services despite being qualified for genetic evaluation. *Journal of Genetic Counseling, 30*(4), 1084-1090.

doi:https://doi.org/10.1002/jgc4.1395.

Erby, Lori H., Wisniewski, Tyler, Lewis, Katie L., Hernandez, Christian, Biesecker, Leslie G., & Biesecker, Barbara B. (2021). Adaptation of the working alliance inventory for the assessment of the therapeutic alliance in genetic counseling. *Journal of Genetic Counseling, 30*(1), 11-21. doi:https://doi.org/10.1002/jgc4.1378

Freeman, Abigail A., Arbuckle, Jacquelynn, & Petty, Elizabeth M. (2021). Preparing genetic counselors to serve Native American communities. *Journal of Genetic Counseling, 30*(5), 1388-1398. doi:https://doi.org/10.1002/jgc4.1405

Godino, Lea, Varesco, Liliana, Bruno, William, Bruzzone, Carla, Battistuzzi, Linda, Franiuk, Marzena, . . . Turchetti, Daniela. (2021). Preferences of Italian patients for return of secondary findings from clinical genome/exome sequencing. *Journal of Genetic Counseling, 30*(3), 665-675. doi:https://doi.org/10.1002/jgc4.1350

Goldman, Jill, Xie, Shanghong, Green, Dina, Naini, Ali, Mansukhani, Mahesh M., & Marder, Karen. (2021). Predictive testing for neurodegenerative diseases in the age of next- generation sequencing. *Journal of Genetic Counseling, 30*(2), 553-562. doi:https://doi.org/10.1002/jgc4.1342

Haddad, Jessica M., Robison, Katina, Beffa, Lindsey, Laprise, Jessica, ScaliaWilbur, Jennifer, Raker, Christina A., . . . Stuckey, Ashley. (2021). Family planning in carriers of BRCA1 and BRCA2 pathogenic variants. *Journal of Genetic Counseling, 30*(6), 1570-1581. doi:https://doi.org/10.1002/jgc4.1423

Hammond, Jennifer, Klapwijk, Jasmijn E., Hill, Melissa, Lou, Stina, Ormond, Kelly E., Diderich, Karin E. M., . . . Lewis, Celine. (2021). Parental experiences of uncertainty following an abnormal fetal anomaly scan: Insights using Han’s taxonomy of uncertainty. *Journal of Genetic Counseling, 30*(1), 198-210.

doi:https://doi.org/10.1002/jgc4.1311.

Haw, Tabitha, & Henriques, Sasha. (2021). Exploring how mothers of a child with a genetic disorder experience their couple relationship in a low socio-economic setting. *Journal of Genetic Counseling, 30*(3), 885-899. doi:https://doi.org/10.1002/jgc4.1391

Hinshaw, Jesse C., Zhao, Lue-Ping, Brimm, John E., Payne, Thomas H., & Hisama, Fuki M. (2021). Value of a genetics clinic evaluation in identifying women at risk for hereditary breast-ovarian cancer syndrome. *Journal of Genetic Counseling, 30*(6), 1591-1597. doi:https://doi.org/10.1002/jgc4.1425

Hoell, Christin, Aufox, Sharon, Nashawaty, Nora, Myers, Melanie F., & Smith, Maureen E. (2021). Comprehension and personal value of negative non-diagnostic genetic panel testing. *Journal of Genetic Counseling, 30*(2), 418-427.

doi:https://doi.org/10.1002/jgc4.1327

Holland, Lucy, Young, Mary-Anne, Lewin, Jeremy, Pearce, Angela, & Thompson, Kate. (2021). Education in youth-friendly genetic counseling. *Journal of Genetic Counseling, 30*(4), 1133-1142. doi:https://doi.org/10.1002/jgc4.1397

Hsieh, Vicky, Braid, Tamara, Gordon, Erynn, & Hercher, Laura. (2021). Direct-to-consumer genetic testing companies tell their customers to ‘see a genetic counselor'. How do genetic counselors feel about direct-to-consumer genetic testing? *Journal of Genetic Counseling, 30*(1), 191-197. doi:https://doi.org/10.1002/jgc4.1310

Hui, Vivian C. C., Li, H. C., Chow, Josh H. K., Ng, Chris S. C., Lui, Constance Y. W., Fung, Jasmine L. F., . . . Lau, Kui Kai. (2021). Understanding and perception of direct-to- consumer genetic testing in Hong Kong. *Journal of Genetic Counseling, 30*(6), 1640- 1648. doi:https://doi.org/10.1002/jgc4.1430

Hurtado-de-Mendoza, Alejandra, Gómez-Trillos, Sara, Graves, Kristi D., Carrera, Pilar, Campos, Claudia, Anderson, Lyndsay, . . . Sheppard, Vanessa B. (2021). Process evaluation of a culturally targeted video for Latinas at risk of hereditary breast and ovarian cancer. *Journal of Genetic Counseling, 30*(3), 730-741. doi:https://doi.org/10.1002/jgc4.1361

Isley, Lauren J., Chamberlain, Aleisha K., Callum, Pamela, & Shamonki, Jaime. (2021). Comparison of methodologies to detect hemoglobinopathy carriers in a multi-ethnic sperm donor population. *Journal of Genetic Counseling, 30*(5), 1399-1406. doi:https://doi.org/10.1002/jgc4.1406

Jacobs, Chris, & McEwen, Alison. (2021). Adapting to the challenges of the global pandemic on genetic counselor education: Evaluating students’ satisfaction with virtual clinical experiences. *Journal of Genetic Counseling, 30*(4), 1074-1083. doi:https://doi.org/10.1002/jgc4.1490

Jacobs, Michelle F., O'Connor, Bridget C., Weldetsadik, Abate Yeshidinber, Tekleab, Atnafu Mekonnen, Bekele, Delayehu, Hanson, Erika, & Quinonez, Shane C. (2021). Knowledge and attitudes about genetic counseling in patients at a major hospital in Addis Ababa, Ethiopia. *Journal of Genetic Counseling, 30*(2), 544-552. doi:https://doi.org/10.1002/jgc4.1340

Jayaraman, Susheela, Koenig, Shelby, Fiddler, Morry, Simi, Eve, Goldenberg, Aaron, Magasi, Susan, & Wicklund, Catherine. (2021). Prenatal genetic counselors' perceptions of the impact of abortion legislation on counseling and access in the United States. *Journal of Genetic Counseling, 30*(6), 1671-1682. doi:https://doi.org/10.1002/jgc4.1433

Juarez, Olivia A., Pencheva, Bojana B., Bellcross, Cecelia, Schneider, Kami W., Turner, Joyce, & Porter, Christopher C. (2021). Cancer genetic counseling for childhood cancer predisposition is associated with improved levels of knowledge and high satisfaction in parents. *Journal of Genetic Counseling, 30*(3), 710-719.

doi:https://doi.org/10.1002/jgc4.1357.

Kahn, Eva, Sheldon, Jane P., Carmichael, Alicia, & Yashar, Beverly M. (2021). Graduate training during the COVID-19 pandemic: North American genetic counseling students' challenges, intolerance of uncertainty, and psychological well-being. *Journal of Genetic Counseling, 30*(5), 1325-1335. doi:https://doi.org/10.1002/jgc4.1501

Kashmola-Perez, Iman, McCarthy Veach, Patricia, Schema, Lynn, & Redlinger-Grosse, Krista. (2021). Genetic counselor use of self-involving responses in a clinical setting: A qualitative investigation. *Journal of Genetic Counseling, 30*(6), 1598-1612. doi:https://doi.org/10.1002/jgc4.1426

Kengne Kamga, Karen, De Vries, Jantina, Nguefack, Séraphin, Munung, Nchangwi Syntia, & Wonkam, Ambroise. (2021). Explanatory models for the cause of Fragile X Syndrome in rural Cameroon. *Journal of Genetic Counseling, 30*(6), 1727-1736. doi:https://doi.org/10.1002/jgc4.1440

Kessler, Lisa Jay, LaMarra, Denise, MacFarlane, Ian M., Heller, Melissa, & Valverde, Kathleen D. (2021). Characterizing standardized patients and genetic counseling graduate education. *Journal of Genetic Counseling, 30*(2), 493-502. doi:https://doi.org/10.1002/jgc4.1335

Khan, Ambreen, Cohen, Stephanie, Weir, Charlene, & Greenberg, Samantha. (2021). Implementing innovative service delivery models in genetic counseling: a qualitative analysis of facilitators and barriers. *Journal of Genetic Counseling, 30*(1), 319-328. doi:https://doi.org/10.1002/jgc4.1325

Kieke, Michele C., Conta, Jessie H., Riley, Jacquelyn D., & Zetzsche, Lindsay H. (2021). The current landscape of genetic test stewardship: A multi-center prospective study. *Journal of Genetic Counseling, 30*(4), 1203-1210. doi:https://doi.org/10.1002/jgc4.1403

Kittikoon, Siraphob, Pithukpakorn, Manop, & Pramyothin, Pornpoj. (2021). Physician awareness, preparedness, and opinions toward consumer-initiated genetic testing in

Thailand: Views from a changing landscape. *Journal of Genetic Counseling, 30*(6), 1535-1543. doi:https://doi.org/10.1002/jgc4.1420

Lee, Daphne S. C., Meiser, Bettina, Mariapun, Shivaani, Hassan, Tiara, Yip, Cheng-Har, Mohd Taib, Nur A., . . . Yoon, Sook-Yee. (2021). Communication about positive BRCA1 and BRCA2 genetic test results and uptake of testing in relatives in a diverse Asian setting. *Journal of Genetic Counseling, 30*(3), 720-729. doi:https://doi.org/10.1002/jgc4.1360

Lee, Heewon, Vogel, Rachel I., LeRoy, Bonnie, & Zierhut, Heather A. (2021). Adult adoptees and their use of direct-to-consumer genetic testing: Searching for family, searching for health. *Journal of Genetic Counseling, 30*(1), 144-157.

doi:https://doi.org/10.1002/jgc4.1304

Leighton, Sarah, Forrest, Laura E., Young, Mary-Anne, Delatycki, Martin B., & Lynch, Elly. (2021). Social media usage in family communication about genetic information: ‘I no longer speak with my sister but she needed to know’. *Journal of Genetic Counseling, 30*(1), 180-190. doi:https://doi.org/10.1002/jgc4.1307

Lepard Tassin, Tiffany, Seraji, Kynoosh, Simonson, Melinda, & Chou, Carrie. (2021). Exploring genetic counselors’ use of pedigree symbols to represent assisted reproductive technology. *Journal of Genetic Counseling, 30*(6), 1773-1778.

doi:https://doi.org/10.1002/jgc4.1434

Lisi, Emily C., & Ali, Nadia. (2021). Opinions of adults affected with later-onset lysosomal storage diseases regarding newborn screening: A qualitative study. *Journal of Genetic Counseling, 30*(6), 1544-1558. doi:https://doi.org/10.1002/jgc4.1421

Liu, Yi, Calzone, Kathleen, Fasaye, Grace-Ann, & Quillin, John. (2021). CDH1 variants leading to gastric cancer risk management decision-making experiences in emerging adults: ‘I am not ready yet’. *Journal of Genetic Counseling, 30*(4), 1091-1104. doi:https://doi.org/10.1002/jgc4.1393.

Lou, Stina, Jensen, Amalie Hahn, & Vogel, Ida. (2021). How are uncertain prenatal genetic results perceived and managed two years after they were received? A qualitative interview study. *Journal of Genetic Counseling, 30*(4), 1191-1202. doi:https://doi.org/10.1002/jgc4.1402

Loudon, Elizabeth, Scott, Stuart A., Rigobello, Robert, Scott, Erick R., Zinberg, Randi, & Naik, Hetanshi. (2021). Pharmacogenomic education among genetic counseling training programs in North America. *Journal of Genetic Counseling, 30*(5), 1500-1508. doi:https://doi.org/10.1002/jgc4.1417

Love-Nichols, Jamie, Uhlmann, Wendy R., Arscott, Patricia, Willer, Cristen, Hornsby, Whitney, & Roberts, J. Scott. (2021). A survey of aortic disease biorepository participants’ preferences for return of research genetic results. *Journal of Genetic Counseling, 30*(3), 645-655. doi:https://doi.org/10.1002/jgc4.1341

Lynce, Filipa, Schlam, Ilana, Geng, Xue, Peshkin, Beth N., Friedman, Sue, Dutil, Julie, . . . Graves, Kristi D. (2021). BRCA1/2 mutations and risk-reducing bilateral salpingo- oophorectomy among Latinas: The UPTAKE study. *Journal of Genetic Counseling, 30*(2), 383-393. doi:https://doi.org/10.1002/jgc4.1322

Lynch, Fiona, Nisselle, Amy, Gaff, Clara L., & McClaren, Belinda. (2021). Rapid acute care genomics: Challenges and opportunities for genetic counselors. *Journal of Genetic Counseling, 30*(1), 30-41. doi:https://doi.org/10.1002/jgc4.1362

Ma, Daria, Ahimaz, Priyanka R., Mirocha, James M., Cook, Lola, Giordano, Jessica L., Mohan, Pooja, & Cohen, Stephanie A. (2021). Clinical genetic counselor experience in the adoption of telehealth in the United States and Canada during the COVID-19 pandemic. *Journal of Genetic Counseling, 30*(5), 1214-1223.

doi:https://doi.org/10.1002/jgc4.1516

Macarov, Michal, Schneider, Nina, Eilat, Avital, & Yahalom, Claudia. (2021). Genetic counseling practice for inherited eye diseases in an Israeli medical center during the COVID-19 pandemic. *Journal of Genetic Counseling, 30*(4), 969-973. doi:https://doi.org/10.1002/jgc4.1479

MacFarlane, Ian, Johnson, Amber, & Zierhut, Heather. (2021). Changes to the genetic counseling workforce as a result of the COVID-19 pandemic. *Journal of Genetic Counseling, 30*(5), 1244-1256. doi:https://doi.org/10.1002/jgc4.1488

Magness, Emily, Magoulas, Pilar, Moscarello, Tia, Ma, Daria, Helm, Benjamin M., & Mizerik, Elizabeth. (2021). Characterization of genetic counselor practices in inpatient care settings. *Journal of Genetic Counseling, 30*(4), 1181-1190.

doi:https://doi.org/10.1002/jgc4.1401

Makhnoon, Sukh, Mork, Maureen, Arun, Banu, Volk, Robert J., & Peterson, Susan K. (2021). Perceptions of provider’s epistemic authority in response to variant of uncertain significance-related recommendations. *Journal of Genetic Counseling, 30*(2), 513-521. doi:https://doi.org/10.1002/jgc4.1337

Mann, Caitlin, Goodhue, Brighton, Guillard, Arianna, Slamon, Jill, Newman, Randa, Zhao, Zhiguo, Dudek, Martha. (2021). The COVID-19 pandemic and reproductive genetic

counseling: Changes in access and service delivery at an academic medical center in the United States. *Journal of Genetic Counseling, 30*(4), 958-968.

doi:https://doi.org/10.1002/jgc4.1462

Martins, Raquel Gomes, & Carvalho, Irene Palmares. (2021). Genetic testing for pheochromocytoma and paraganglioma: SDHx carriers’ experiences. *Journal of Genetic Counseling, 30*(3), 872-884. doi:https://doi.org/10.1002/jgc4.1390

Marzulla, Tessa, Roberts, J. Scott, DeVries, Raymond, Koeller, Diane R., Green, Robert C., & Uhlmann, Wendy R. (2021). Genetic counseling following direct-to consumer genetic testing: Consumer perspectives. *Journal of Genetic Counseling, 30*(1), 329-334. doi:https://doi.org/10.1002/jgc4.1309

Mauer, Caitlin, Zimmerman, John, Lahiri, Sayoni, Watson, Elise, Parsi, Lily, Berg, Jordan, & Pirzadeh-Miller, Sara. (2021). Adapting genetic counseling operations amidst the COVID-19 pandemic. *Journal of Genetic Counseling, 30*(4), 949-955. doi:https://doi.org/10.1002/jgc4.1474

McGuinness, Molly, Fassi, Emily, Wang, Catharine, Hacking, Claire, & Ellis, Victoria. (2021). Breast cancer polygenic risk scores in the clinical cancer genetic counseling setting: Current practices and impact on patient management. *Journal of Genetic Counseling, 30*(2), 588-597. doi:https://doi.org/10.1002/jgc4.1347

Meadows, Rachel J., & Padamsee, Tasleem J. (2021). Financial constraints on genetic counseling and further risk-management decisions among U.S. women at elevated breast cancer risk. *Journal of Genetic Counseling, 30*(5), 1452-1467. doi:https://doi.org/10.1002/jgc4.1413

Mendes, Álvaro, Sequeiros, Jorge, & Clarke, Angus J. (2021). Between responsibility and desire: Accounts of reproductive decisions from those at risk for or affected by late- onset neurological diseases. *Journal of Genetic Counseling, 30*(5), 1480-1490. doi:https://doi.org/10.1002/jgc4.1415

Menke, Chelsea, Nagaraj, Chinmayee B., Dawson, Brian, He, Hua, Tawde, Sanjukta, & Wakefield, Emily G. (2021). Understanding and interpretation of a variant of uncertain significance (VUS) genetic test result by pediatric providers who do not specialize in genetics. *Journal of Genetic Counseling, 30*(6), 1559-1569.

doi:https://doi.org/10.1002/jgc4.1422

Mills, Rachel, MacFarlane, Ian M., Caleshu, Colleen, Ringler, Margaret A., & Zierhut, Heather

A. (2021). Genetic counselor experiences with telehealth before and after COVID-19.

*Journal of Genetic Counseling, 30*(4), 999-1009. doi:https://doi.org/10.1002/jgc4.1465 Milo Rasouly, Hila, Cuneo, Nicole, Marasa, Maddalena, DeMaria, Natalia, Chatterjee,

Debanjana, Thompson, Jacqueline J., . . . Gharavi, Ali G. (2021). GeneLiFT: A novel test to facilitate rapid screening of genetic literacy in a diverse population undergoing genetic testing. *Journal of Genetic Counseling, 30*(3), 742-754. doi:https://doi.org/10.1002/jgc4.1364

Mueller, Rebecca, Schindewolf, Erica, Williams, Samantha, & Jay Kessler, Lisa. (2021). ‘Steep learning curves’ to ‘Smooth Sailing’: A reappraisal of telegenetics amidst the COVID-19 pandemic. *Journal of Genetic Counseling, 30*(4), 1010-1023. doi:https://doi.org/10.1002/jgc4.1487

Page, Robin L., Murphey, Christina, Aras, Yahyahan, Chen, Lei-Shih, & Loftin, Ryan. (2021). Pregnant Hispanic women's views and knowledge of prenatal genetic testing. *Journal of Genetic Counseling, 30*(3), 838-848. doi:https://doi.org/10.1002/jgc4.1383

Pan, Vivian, Doerr, Megan, Hoell, Christin, Ryan, Lauren, Erwin, Deanna J., Hooker, Gillian,

. . . The National Society of Genetic Counselors, G. C. Sars-CoV-Impact Survey Working Group. (2021). Results of the Genetic Counselor SARS-CoV-2 Impact Survey from the National Society of Genetic Counselors: Progress and penalty during the COVID-19 pandemic. *Journal of Genetic Counseling, 30*(4), 989-998. doi:https://doi.org/10.1002/jgc4.1484

Park, Min Seon, Weissman, Scott M., Postula, Kristen J. Vogel, Williams, Carmen S., Mauer, Caitlin B., & O’Neill, Suzanne M. (2021). Utilization of breast cancer risk prediction models by cancer genetic counselors in clinical practice predominantly in the United States. *Journal of Genetic Counseling, 30*(6), 1737-1747.

doi:https://doi.org/10.1002/jgc4.1442

Plamann, Katie, McCarthy Veach, Patricia, LeRoy, Bonnie S., MacFarlane, Ian M., Petzel, Sue V., & Zierhut, Heather A. (2021). Effects of monitoring versus blunting on the public’s preferences for information in a hypothetical cancer diagnosis scenario. *Journal of Genetic Counseling, 30*(1), 132-143. doi:https://doi.org/10.1002/jgc4.1302

Quirin, Kayla, Hines, Karrie A., & Wetherill, Leah. (2021). Genetic counseling for advanced paternal age: A survey of genetic counselors' current practice. *Journal of Genetic Counseling, 30*(2), 428-438. doi:https://doi.org/10.1002/jgc4.1328

Rao, Nivedita, Kanago, Dhanashree, Morris, Monisha, Narayan, Vinu, Varshney, Kruti, Gn, Sanjeeva, & Bhat, Meenakshi. (2021). Telegenetics: The experience of an Indian center (Centre for Human Genetics) during the COVID-19 pandemic. *Journal of Genetic Counseling, 30*(5), 1224-1232. doi:https://doi.org/10.1002/jgc4.1517

Redlinger-Grosse, Krista, MacFarlane, Ian M., Cragun, Deborah, & Zierhut, Heather. (2021). A Delphi study to prioritize genetic counseling outcomes: What matters most. *Journal of Genetic Counseling, 30*(3), 676-692. doi:https://doi.org/10.1002/jgc4.1352

Rezich, Brianna M. Z., Malone, Jaime A., Reiser, Gwen, Zimmerman, Holly H., Blase, Terri L., & Fishler, Kristen P. (2021). Telehealth genetic services during the COVID-19 Pandemic: Implementation and patient experiences across multiple specialties in Nebraska. *Journal of Genetic Counseling, 30*(5), 1233-1243.

doi:https://doi.org/10.1002/jgc4.1507

Richards, Jonica L., Petty, Elizabeth M., & Zelenski, Amy. (2021). Exploring empathy in genetic counseling students and new genetic counselors. *Journal of Genetic Counseling, 30*(1), 293-304. doi:https://doi.org/10.1002/jgc4.1321

Riggan, Kirsten A., Gross, Brianna, Close, Sharron, Weinberg, Abigail, & Allyse, Megan A. (2021). Prenatal Genetic Diagnosis of a Sex Chromosome Aneuploidy: Parent Experiences. *Journal of Genetic Counseling, 30*(5), 1407-1417. doi:https://doi.org/10.1002/jgc4.1407

Rolf, Bradley, Blue, Elizabeth E., Bucks, Stephanie, Dorschner, Michael O., & Jayadev, Suman. (2021). Genetic counseling for early onset and familial dementia: Patient perspectives on exome sequencing. *Journal of Genetic Counseling, 30*(3), 793-802. doi:https://doi.org/10.1002/jgc4.1379

Rovira-Moreno, Eulàlia, Abuli, Anna, Codina-Sola, Marta, Valenzuela, Irene, Serra-Juhe, Clara, Cuscó, Ivon, . . . Tizzano, Eduardo F. (2021). Beyond the disease itself: A cross- cutting educational initiative for patients and families with rare diseases. *Journal of Genetic Counseling, 30*(3), 693-700. doi:https://doi.org/10.1002/jgc4.1354

Ruderman, Maggie, Berro, Tala, Torrey Sosa, Lillian, & Zayhowski, Kimberly. (2021). Genetic counselors' experiences with transgender individuals in prenatal and preconception settings. *Journal of Genetic Counseling, 30*(4), 1105-1118. doi:https://doi.org/10.1002/jgc4.1394

Schlub, Georgina M., Crook, Ashley, Barlow-Stewart, Kristine, Fleming, Jane, Kirk, Judy, Tucker, Kathy, & Greening, Sian. (2021). Helping young children understand inherited cancer predisposition syndromes using bibliotherapy. *Journal of Genetic Counseling, 30*(4), 1119-1132. doi:https://doi.org/10.1002/jgc4.1396

Schmanski, Andrew, Roberts, Emily, Coors, Marilyn, Wicks, Stephen J., Arbet, Jaron, Weber, Rachel, . . . Taylor, Matthew R. G. (2021). Research participant understanding and engagement in an institutional, self-consent biobank model. *Journal of Genetic Counseling, 30*(1), 257-267. doi:https://doi.org/10.1002/jgc4.1316

Schwaninger, Gunda, Benjamin, Caroline, Rudnik-Schöneborn, Sabine, & Zschocke, Johannes. (2021). The genetic counseling profession in Austria: Stakeholders’ perspectives. *Journal of Genetic Counseling, 30*(3), 861-871.

doi:https://doi.org/10.1002/jgc4.1389

Shannon, Kristen M., Emmet, Margaret M., Rodgers, Linda H., Wooters, Mackenzie, & Seidel, Meredith L. (2021). Transition to telephone genetic counseling services during the COVID-19 pandemic. *Journal of Genetic Counseling, 30*(4), 984-988.

doi:https://doi.org/10.1002/jgc4.1365

Sim, Jackie, Shaw, Tarryn, Li, Shao-Tzu, Courtney, Eliza, Yuen, Jeanette, Chiang, Jianbang, .

. . Ngeow, Joanne. (2021). Understanding patients' views and willingness toward the use of telehealth in a cancer genetics service in Asia. *Journal of Genetic Counseling, 30*(6), 1658-1670. doi:https://doi.org/10.1002/jgc4.1432

Similuk, Morgan N., Yan, Jia, Setzer, Michael R., Jamal, Leila, Littel, Patricia, Lenardo, Michael, & Su, Helen C. (2021). Exome sequencing study in a clinical research setting finds general acceptance of study returning secondary genomic findings with little decisional conflict. *Journal of Genetic Counseling, 30*(3), 766-773.

doi:https://doi.org/10.1002/jgc4.1367

Smith, Cayleen, Hashmi, Syed S., Czerwinski, Jennifer, Wagner, Victoria F., Promecene, Pamela, Milentijevic, Irena, & Ramdaney, Aarti. (2021). The impact of genetic counseling on women's grief and coping following termination of pregnancy for fetal anomaly. *Journal of Genetic Counseling, 30*(2), 522-532.

doi:https://doi.org/10.1002/jgc4.1338

Speechly, Catherine, Stenhouse, Rachael, Berman, Yemima, Barlow-Stewart, Kristine, Fleming, Jane, Petrie, Dianne, & Culling, Bronwyn. (2021). Genetic counselors, patients', and carers’ views on an Australian clinical genetics service information

system. *Journal of Genetic Counseling, 30*(5), 1440-1451.

doi:https://doi.org/10.1002/jgc4.1412

Stachowiak, Samantha, Jacquart, Amanda, Zimmermann, Michael T., George, Ben, Dong, Huaying, & Geurts, Jennifer L. (2021). Germline evaluation of patients undergoing tumor genomic profiling: An academic cancer center’s experience with implementing a germline review protocol. *Journal of Genetic Counseling, 30*(3), 900-910.

doi:https://doi.org/10.1002/jgc4.1392

Stallman, Chris, Kieran, Shannon, Quinn, Dorothy, & Schaibley, Valerie M. (2021). Rethinking genetic counseling clinical skills training in the time of COVID-19. *Journal of Genetic Counseling, 30*(5), 1310-1315. doi:https://doi.org/10.1002/jgc4.1503

Stoddard, Andrea, McCarthy Veach, Pat, MacFarlane, Ian M., LeRoy, Bonnie, & Tryon, Rebecca. (2021). Genetic counseling student demographics: an empirical comparison of two cohorts. *Journal of Genetic Counseling, 30*(1), 211-228.

doi:https://doi.org/10.1002/jgc4.1312

Studwell, Courtney M., Kelley, Emily G., Undiagnosed Diseases, Network, Sinsheimer, Janet S., Palmer, Christina G. S., & LeBlanc, Kimberly. (2021). Family genetic result communication in rare and undiagnosed disease communities: Understanding the practice. *Journal of Genetic Counseling, 30*(2), 439-447.

doi:https://doi.org/10.1002/jgc4.1329

Stuttgen, Kelsey, McCague, Allison, Bollinger, Juli, Dvoskin, Rachel, & Mathews, Debra. (2021). Whether, when, and how to communicate genetic risk to minors: ‘I wanted more information but I think they were scared I couldn’t handle it’. *Journal of Genetic Counseling, 30*(1), 237-245. doi:https://doi.org/10.1002/jgc4.1314

Sulmonte, Laura A. G., Bisordi, Katharine, Ulm, Elizabeth, & Nusbaum, Rachel. (2021). Open communication of Duchenne muscular dystrophy facilitates disclosure process by parents to unaffected siblings. *Journal of Genetic Counseling, 30*(1), 246-256. doi:https://doi.org/10.1002/jgc4.1315

Thom, Jamey, & Haw, Tabitha. (2021). Awareness of genetic counseling services among allied healthcare professionals in South Africa. *Journal of Genetic Counseling, 30*(6), 1649- 1657. doi:https://doi.org/10.1002/jgc4.1431

Ting, Michael Sing Onn, Clarke, Angus, & McAllister, Marion. (2021). Assessing sensitivity to change of the genomics outcome scale (GOS). *Journal of Genetic Counseling, 30*(6), 1767-1772. doi:https://doi.org/10.1002/jgc4.1429

Triebold, Malia, Skov, Karina, Erickson, Lindsay, Olimb, Sarah, Puumala, Susan, Wallace, Ian, & Stein, Quinn. (2021). Geographical analysis of the distribution of certified genetic counselors in the United States. *Journal of Genetic Counseling, 30*(2), 448-456. doi:https://doi.org/10.1002/jgc4.1331

Turchetti, Daniela, Battistuzzi, Linda, Bertonazzi, Benedetta, & Godino, Lea. (2021). Sudden shift to remote genetic counseling during the COVID-19 pandemic: Experiences of genetics professionals in Italy. *Journal of Genetic Counseling, 30*(4), 1024-1037. doi:https://doi.org/10.1002/jgc4.1441

Underhill-Blazey, Meghan, Blonquist, Traci, Chittenden, Anu, Pozzar, Rachel, Nayak, Manan, Lansang, Kristina, . . . Stopfer, Jill E. (2021). Informing models of cancer genetics care in the era of multigene panel testing with patient-led recommendations. *Journal of Genetic Counseling, 30*(1), 268-282. doi:https://doi.org/10.1002/jgc4.1317

VanDyke, Rebecca E., Hashimoto, Sayaka, Morales, Ana, Pyatt, Robert E., & Sturm, Amy C. (2021). Impact of variant reclassification in the clinical setting of cardiovascular genetics. *Journal of Genetic Counseling, 30*(2), 503-512.

doi:https://doi.org/10.1002/jgc4.1336

Verbrugge, Jennifer, Cook, Lola, Miller, Mandy, Rumbaugh, Malia, Schulze, Jeanine, Heathers, Laura, . . . Foroud, Tatiana. (2021). Outcomes of genetic test disclosure and genetic counseling in a large Parkinson's disease research study. *Journal of Genetic Counseling, 30*(3), 755-765. doi:https://doi.org/10.1002/jgc4.1366

Wagner, Chelsea, Stein, Quinn, & Singletary, Claire N. (2021). Genesurance counseling: Current training practices of genetic counseling graduate programs in the United States. *Journal of Genetic Counseling, 30*(6), 1757-1766.doi:https://doi.org/10.1002/jgc4.1444

Wagner, Naomi E., Witherington, Sarah, Waldman, Larissa, Ryan, Lauren, & Hardy, MelanieW. (2021). Analysis of the reported use of practice-based competencies by North American genetic counselors during the COVID-19 pandemic. *Journal of Genetic Counseling, 30*(5), 125-1268. doi:https://doi.org/10.1002/jgc4.1504

Walsh, Matthew B., Charen, Krista, Shubeck, Lisa, McConkie-Rosell, Allyn, Ali, Nadia, Bellcross, Cecelia, & Sherman, Stephanie L. (2021). Men with an FMR1 premutation and their health education needs. *Journal of Genetic Counseling, 30*(4), 1156-1167. doi:https://doi.org/10.1002/jgc4.1399

Wang, Qun, Pang, Cong, Meng, Lingyu, & Wang, Guoyu. (2021). Public perceived knowledge of, attitude toward, and use of genetic testing in urban China. *Journal of Genetic Counseling, 30*(6), 1629-1639. doi:https://doi.org/10.1002/jgc4.1428

Warias, Ashley, Ferguson, Meghan, Chamberlain, Erin, Currie, Lauren, Snow, Nicole, Matheson, Kara, Kieser, Katharina. (2021). Universal access to genetic counseling for women with epithelial ovarian cancer in Nova Scotia: Evaluating a new collaborative care model. *Journal of Genetic Counseling, 30*(5), 1491-1499. doi:https://doi.org/10.1002/jgc4.1416

Wessels, Tina-Marié, Düsterwald, Gillian, Barlow, Robyn, Cameron-Mackintosh, Sinead, Diedericks, Angelique, Francois, Sydney, . . . Vorster, Nina. (2021). Genetic counseling experiences at the University of Cape Town during COVID-19. *Journal of Genetic Counseling, 30*(5), 1298-1309. doi:https://doi.org/10.1002/jgc4.1520

Willis, Amanda M., Smith, Sian K., Meiser, Bettina, James, Paul A., Ballinger, Mandy L., Thomas, David M., . . . Young, Mary-Anne. (2021). Influence of lived experience on risk perception among women who received a breast cancer polygenic risk score: ‘Another piece of the pie’. *Journal of Genetic Counseling, 30*(3), 849-860. doi:https://doi.org/10.1002/jgc4.1384

Yusuf, Afiqah, Peltekova, Iskra, Savion-Lemieux, Tal, Frei, Jennifer, Joober, Ridha, Howe, Jennifer, . . . Elsabbagh, Mayada. (2021). Adaptation and validation of the Genetic Counseling Outcome Scale for autism spectrum disorders and related conditions. *Journal of Genetic Counseling, 30*(1), 305-318. doi:https://doi.org/10.1002/jgc4.1323

Zalan, Alice, Maga, Tara, Perpich, Melody, Pillay Smiley, Natasha, & Weiss McQuaid, Shelly. (2021). Parental attitudes regarding the need for genetic services in a pediatric brain tumor survivorship program. *Journal of Genetic Counseling, 30*(2), 533-543. doi:https://doi.org/10.1002/jgc4.1339

Zayts-Spence, Olga, Fung, Jasmine L. F., & Chung, Brian H. Y. (2021). ‘Do language and culture really matter?’: A trans-disciplinary investigation of cultural diversity in genetic counseling in Hong Kong. *Journal of Genetic Counseling, 30*(1), 75-84. doi:https://doi.org/10.1002/jgc4.1385

Zhao, Shixi, Chen, Wei-Ju, Dhar, Shweta U., Eble, Tanya N., Kwok, Oi-Man, & Chen, Lei- Shih. (2021). Pursuing genetic testing for children with autism spectrum disorders: What do parents think? *Journal of Genetic Counseling, 30*(2), 370-382. doi:https://doi.org/10.1002/jgc4.1320

**Supplemental Note 2: Inter-coder reliability**

Inter-coder reliability was *moderate* for the “discussion of implications of REA reporting for genetic counseling practice” variable (85.71%; k = 0.417; s.e. = 0.189), *substantial* for the following variables: “reference to REA factors in the interpretation of results” variable (92.86%; k = 0.708; s.e. = 0.189), “definition of REA factors” (96.30%; k = 0.649; s.e = 0.180), “use of REA factors in data analysis” (82.14%; k = 0.713; s.e. = 0.139), and “inclusion of REA factors in the discussion of study limitations” (89.29%; k = 0.781: s.e. = 0.184) and a*lmost perfect* for the following variables: “inclusion of REA factors in sampling procedures” (96.43%; k = 0.887; s.e. = 0.188), “description of participants’ REA characteristics ascertainment method” (89.29%; k = 0.834; s.e. = 0.127), “reporting of match or mismatch of interviewers’ and participants’ REA characteristics” (92.86%; k = 0.857: s.e. = 0.178). Interpretation of k is the following: < 0 = *poor*, 0.0 – 0.20 = *slight*, 0.21 – 0.40 = *fair*, 0.41 – 0.60 = *moderate*, 0.61 – 0.80 = *substantial*, 0.81 – 1.00 = *almost perfect.*

**Supplemental Note 3: Group labels used under the population descriptors “race/ethnicity” or “race and ethnicity” or “race and/or ethnicity”**

| African American; African American/Black; Alaskan; American Chinese; American Indian;  American Indian or Alaskan Native or First Nations; American Indian or Alaskan; American Indian/Alaska Native; Asian; Asian American; Asian Indian; Asian or East/Southeast Asian or Asian Indian; Asian, non-Hispanic; Asian/Pacific Islander; Biracial; Caribbean; Black; Black African American or African; Black or African American; Black or African American, Hispanic; Black or African American, non-Hispanic; Black, non-Hispanic; Chinese; Desi; Filipino; First-generation; Guyanese-American; Hispanic; Hispanic Latino or Spanish; Hispanic or Latino(a); Hispanic or Latinx; Indian; Indian-American; Iraqi; Jewish; Latina; Mexican; Middle Easter or North African; Middle Eastern; Mixed Race; Multiple Race/Ethnicities; Multiracial; Muslim; Native American; Native Hawaiian or other Pacific Islander; Native Hawaiian or Pacific Islander; Nepali Brahmin; Non-Hispanic White; Non-White; Other race, Hispanic; Other race, non-Hispanic; Other, non-Hispanic (including multiple races); Pakistani; Pakistani-American; South Asian; Southeast Asian; Two or more races; West Indian; White; White or Caucasian; White, Hispanic; White, non-Hispanic; 1.5 generation. |
| --- |

## Supplemental Note 4: Extended quote from Carmichael et al. (2021)

When asked to select the NSGC racial or ethnic category with which they had ‘the most in common’, they responded as follows: Asian (n = 12), Asian Indian (n = 11), Hispanic or Latino (n = 7), White or Caucasian (n = 7), Black or African American (n = 4), and Other (n = 2). Participants were able to select more than one category; those who selected ‘White or Caucasian’ were all multi-racial. It is noteworthy that when asked to describe their race or ethnicity in their own words, participants offered a much broader range of categories: their responses included African American/Black, Asian, American Chinese, Biracial, Caribbean, Chinese, Desi, Filipino, First-generation, Guyanese-American, Hispanic, Indian, Indian-American, Iraqi, Latina, Mexican, Mixed -race, Muslim, Native American, Nepali Brahmin, Pakistani, Pakistani-American, South Asian, Southeast Asian, West Indian, and 1.5 generation. (Carmichael, Redlinger‐ Grosse, & Birnbaum, 2021, pp. 815-816)

**Supplemental Table 1. Items and instructions of the mapping proforma**

| **Item** |  |
| --- | --- |
| 1. | Article title |
| 2. | Author list |
| 3. | Specialty Codes:   1. Cancer genetics 2. Newborn screening 3. Reproductive genetics (Carrier screening, prenatal, PGD) 4. Pediatrics 5. Adult 6. Cross-disciplinary 7. Other (Specify) |
| 4. | Study aim/s |
|  | **REA definition and study rationale** |
| 5. | Define REA factor(s)  *Do the authors provide a definition or conceptualization of REA factors?* Count any definition of REA terms as positive, even if vague or basic (e.g., “we used the 2000 Census categories”).  Codes:   1. YES 2. NO |
| 6. | Discuss the role of REA factors in the rationale for the study  *Do the authors discuss the rationale for the study topic or study design in terms of REA factors?*  Codes:   1. YES 2. NO |
|  | **Methods & Results** |
| 7. | Study design/method Codes:   1. Quantitative 2. Qualitative 3. Mixed-methods |
| 8. | Country/ies where the study took place (Dropdown list of countries) |
| 9. | Characteristics of the sample Codes:   1. Individuals from the general population/community samples/patients 2. Health professionals/researchers/educators/students 3. Participants from both group 1 and group 2 |

| 10. | Include REA factors in sampling procedure Codes:   1. YES 2. NO   Code “positive” (1) only articles that attended specifically to REA factors in sampling (e.g., justified sampling a single REA group, stratified by REA, oversampling of REA groups). Other articles code “negative” (0) (e.g., no  attention paid to REA, article reported sample of convenience without discussing the relevance of REA factors). |
| --- | --- |
| 11. | Describe REA characteristics of the sample  *Do the authors describe their sample in terms of REA variables?*  Codes:   1. YES 2. NO   If oversampling of a specific race/ethnic/ancestry group occurred, code as 1. For instance, if a person needs to self-identify as White to be eligible to  participate in the study and this is clearly outlined, code as 1. |
| 12.  13. | Describe how participants’ REA characteristics were ascertained  Codes:   1. Not specified 2. Self-report 3. Place of birth 4. Assigned by interviewer/physician/researcher 5. Data extraction from medical records 6. Other: 7. NA (answer to question 11 is 0, i.e., REA characteristics of the sample are not described)   Specific method of REA characteristics ascertainment in case of self-report Codes:   1. Self-report free text 2. Self-report from multiple-choice 3. Other: 4. Not reported 5. NA if the answer to variable 12 is 0,2,3,4, 5 or 6 |
| 14. REA population descriptors  Codes:   1. race and ethnicity as a single population descriptor (e.g.: race/ethnicity) 2. race and ethnicity are separate population descriptors (or only one is mentioned) 3. ancestry 4. not applicable because the sample is not described in terms of REA characteristics 5. not specified/unable to tell   15. Group labels used to describe the sample  Note (verbatim) the group labels used by the authors to describe the sample: ________  Code 2 (NA) if the answer to question 11 is NO (0), the sample is not described in terms of REA characteristics | |

| 16. | Report “Other” among group labels  Codes:   1. YES 2. NO 3. NA |
| --- | --- |
| 17. | Mention match or mismatch of interviewers’ and participants’ REA  characteristics  *Do the authors mention the relevance of the REA characteristics of the interviewers vis-à-vis the REA characteristics of the participants?*  Codes:   1. YES 2. NO 3. NA |
| 18. | Used REA factors in data analysis Codes:   1. articles that could have conducted REA-based analyses but did not (e.g., articles that included multiple REA groups) 2. any inclusion of REA factors in analyses including articles that sampled a single REA group with the intent of studying that particular population (examples of analyses are bivariate analyses of the association between REA variables and any outcome variables, analyses of the relationship between non-REA independent and dependent variables adjusted for REA in multiple regression, and analyses of the relationship between REA variables and outcome variables adjusted for non-REA variables in multiple regression). 3. NA (if no REA characteristics of the sample are described, this equates to answer 0 to question 11, or it is a qualitative study, or only one REA group was included in the study but it was a convenience sample) |
| 19. | Perform “White” vs “non-White/non-Caucasian/Other/more than once race” data analysis  Codes:   1. YES 2. NO 3. NA |
|  |  |
|  | **Discussion** |
| 20. | Refer to REA factors in the interpretation of results  *Do the authors refer to any REA factors in the interpretation of their results?*   1. Not at all 2. Somewhat 3. Moderately 4. A great deal   For data analysis purposes dichotomize response options Code “not at all” and “somewhat” as “negative” = code 0; Code “moderate” and “a great deal” as “positive” = code 1.  Codes:   - 1. YES   2. NO |

| 21. | Include REA factors in the discussion of study limitations  *Do the authors discuss/acknowledge the study limitations in terms of REA factors?*  Codes:   1. YES 2. NO |
| --- | --- |
| 22. | Discussion of implications of REA reporting for genetic counseling practice *Do the authors discuss the implications of REA reporting for genetic counseling practice?*  Codes:   1. YES 2. NO |

**Supplemental Table 2: Study genetic counseling specialty**

| **Specialty** | n | % |
| --- | --- | --- |
| Cancer genetics | 27 | 20 |
| Adult genetics | 18 | 14 |
| Reproductive geneticsa | 17 | 13 |
| Pediatrics | 15 | 11 |
| Newborn Screening | 1 | 1 |
| Otherb | 54 | 41 |
| a Prenatal, in-vitro fertilization, carrier screening, preimplantation genetic diagnosis  b Cross-disciplinary (n=33), direct to consumer testing (n=3), Education (n=10), Neurogenetics (n=3), Ophthalmology (n=1), student experience (n=4) | | |

**Supplemental Table 3: Location of studies execution**

| **Study location** | **N (%)** |
| --- | --- |
| North America (including Canada and the USA) | 99 (75%) |
| Europe (including Austria, Denmark, Italy, Portugal, Spain, the Netherlands,  UK) | 11(8%) |
| Australasia (including Australia and New Zealand) | 8 (6%) |
| Asia (including China, Hong Kong, India, Malesia, Singapore, and Thailand) | 7 (5%) |
| Africa (including Cameroon, Ethiopia and South Africa) | 5 (4%) |
| Middle East (including Israel and Oman) | 2 (2%) |

**Supplemental Table 4: Summary of findings**

|  | **Quantitative** | **Qualitative** | **Mix-methods** |
| --- | --- | --- | --- |
|  | **n =86** | **n = 39** | **n = 7** |
|  |  |  |  |
| General population | 51/86 | 24/39 | 5/7 |
| Health professionals | 34/86 | 13/39 | 2/7 |
| General population + Health professionals | 1/86 | 2/39 | 0/7 |
|  |  |  |  |
| **Described participants’ REA characteristics** | **56/86** | **20/39** | **4/7** |
| General population | 40/56 | 15/20 | 3/4 |
| Health professionals | 15/56 | 5/20 | 1/4 |
| General population + Health professionals | 1/56 | 0/20 | 0/4 |
| Provided rationale in terms of REA factors | 11/56 | 6/20 | 1/4 |
| Provided definition of REA | 4/56 | 1/20 | 0/4 |
| Attention to REA factors in sampling | 4/56 | 7/20 | 1/4 |
| Ascertainment of REA characteristics through self-report | 44/56 | 15/20 | 4/4 |
| Mention of match-mismatch of participants and interviewers’ REA | na | 3/20 | 0/3^ |
| Used REA factor in data analyses | 19/55* | na | 2/4 |
| White vs Non-White data analyses | 5/19 | na | 1/2 |
| Refer to REA factors in the interpretation of results | 10/56 | 5/20 | 1/4 |
| Include REA factors in the discussion of study limitations | 24/56 | 10/20 | 3/4 |
| Discussion of implications of REA reporting for genetic counseling practice | 8/56 | 3/20 | 1/4 |
| ^note: variable applicable only to three out of four mixed research studies, since only three studies conducted interviews or focus groups; *note: 55/56 included more than one REA group or recruited participants from a specific REA group with the intent of studying it (i.e., not a convenience sample resulting in a homogenous group of participants with the same REA characteristics); na = not applicable | | | |
